# The causal relationship between human blood metabolites and the risk of visceral obesity: a mendelian randomization analysis

**DOI:** 10.1186/s12944-024-02035-x

**Published:** 2024-02-07

**Authors:** Zhaoxiang Wang, Qichao Yang

**Affiliations:** 1 Department of Endocrinology, Affiliated Kunshan Hospital of Jiangsu University, Kunshan, Jiangsu 215300 China; 2 Department of Endocrinology, Affiliated Wujin Hospital of Jiangsu University, Changzhou, Jiangsu 213017 China; 3 Wujin Clinical College of Xuzhou Medical University, Changzhou, Jiangsu 213017 China

**Keywords:** Visceral obesity, Blood metabolites, GWAS, LDSC, Mendelian randomization analysis

## Abstract

**Background:**

We aimed to explore the causal relationship between blood metabolites and the risk of visceral obesity, as measured by visceral adipose tissue (VAT).

**Methods:**

Summary statistics for 486 blood metabolites and total, as well as sex-stratified, MRI-derived VAT measurements, adjusted for body mass index (BMI) and height, were collected from previous genome-wide association studies (GWAS). A two-sample Mendelian Randomization (MR) design was used. Comprehensive evaluation was further conducted, including sensitivity analysis, linkage disequilibrium score (LDSC) regression, Steiger test, and metabolic pathway analysis.

**Results:**

After multiple testing correction, arachidonate (20:4n6) has been implicated in VAT accumulation (β = 0.35, 95%CI:0.18–0.52, *P* < 0.001; FDR = 0.025). Additionally, several blood metabolites were identified as potentially having causal relationship (FDR < 0.10). Among them, lysine (β = 0.67, 95%CI: 0.28–1.06, *P* < 0.001; FDR = 0.074), proline (β = 0.30, 95%CI:0.13–0.48, *P* < 0.001; FDR = 0.082), valerate (β = 0.50, 95%CI:0.23–0.78, *P* < 0.001, FDR = 0.091) are associated with an increased risk of VAT accumulation. On the other hand, glycine (β=-0.21, 95%CI: -0.33–0.09), *P* < 0.001, FDR = 0.076) have a protective effect against VAT accumulation. Most blood metabolites showed consistent trends between different sexes. Multivariable MR analysis demonstrated the effect of genetically predicted arachidonate (20:4n6) and proline on VAT remained after accounting for BMI and glycated hemoglobin (HbA1c). There is no evidence of heterogeneity, pleiotropy, and reverse causality.

**Conclusion:**

Our MR findings suggest that these metabolites may serve as biomarkers, as well as for future mechanistic exploration and drug target selection of visceral obesity.

**Supplementary Information:**

The online version contains supplementary material available at 10.1186/s12944-024-02035-x.

## Introduction

Obesity is a major global health problem, affecting billions of people worldwide and showing significant heterogeneity [1, 2]. The distribution of adipose tissue was considered as an important factor in determining the health risks of obesity [1]. VAT, the intra-abdominal fat encapsulating internal organs, is metabolically active and potentially harmful, unlike subcutaneous adipose tissue (SAT) [2, 3]. Visceral obesity, characterized by excessive VAT accumulation, is significantly linked to numerous detrimental health conditions, including cardiovascular diseases, metabolic disorders, and cancers [2, 4, 5].

Metabolomics, a modern omics-based technology, has greatly enhanced our understanding of disease mechanisms by uncovering intermediate metabolites and altered metabolic pathways [6]. The analysis of extensive datasets generated by previous metabolomics studies has revealed the vital role of metabolic products, such as lipids, amino acids, and so on, in regulating energy metabolism, lipid synthesis, adipocyte differentiation, lipid oxidation, and insulin sensitivity [7]. Nevertheless, the precise association between blood metabolites and the distribution of adipose tissue remains elusive in the current scientific understanding. Thus, it is imperative to identify metabolites associated with adipose tissue, particularly VAT, to investigate the potential metabolic mechanisms of visceral obesity and develop targeted intervention measures.

Compared to conventional observational studies, the MR approach is a powerful method for obtaining robust evidence of causality utilizing genetic variants as instrumental variables (IVs) to address confounding [8]. In this study, we followed the STROBE-MR guidelines and conducted a two-sample MR analysis to investigate the potential causal association between human blood metabolites and VAT accumulation [9].

## Methods

### Study design

The MR analysis was conducted based on three crucial assumptions: (1) The IVs used in the analysis exhibited a strong association with blood metabolites. (2) The chosen IVs and potential confounding variables, which could impact blood metabolites and VAT, were not interrelated. (3) The IVs solely influenced VAT through their impact on blood metabolites.

### Data sources

Blood metabolite genetic data were obtained from the metabolomics GWAS server (http://metabolomics.helmholtz-muenchen.de/gwas/), or the GWAS Catalog (https://www.ebi.ac.uk/gwas/) [10, 11]. Shin et al. performed a comprehensive GWAS of non-targeted metabolomics, identifying 486 human serum metabolites with genetic influences [11]. A total of 7824 participants were enrolled from two European population cohorts: 1768 participants from the KORA F4 study in Germany and 6056 from the UK Twin Study. Fasting serum samples were analyzed using non-targeted mass spectrometry analysis. Metabolon, Inc. was employed for standardized processes of identification and relative qualification (https://www.metabolon.com/) [12]. A total of 486 metabolites were analyzed, comprising 177 unknown metabolites and 309 known metabolites, which were further classified into eight biochemical classes: peptide, nucleotide, amino acid, energy, cofactors and vitamins, lipid, carbohydrate, and xenobiotics. On the other hand, the GWAS summary statistics for total and sex-stratified VAT utilized MRI scans, including 20,038 women and 19,038 men, were obtained from the UK Biobank (https://cvd.hugeamp.org/) [13]. These scans were annotated using deep learning techniques. The GWAS was conducted using the UK Biobank imputed genotypes version 3, excluding SNPs with a minor allele frequency less than 1% and imputation quality below 0.9. The GWAS analysis was performed using BOLT-LMM v2.3.4. To account for potential confounding factors, the measures were adjusted for BMI, height, age at the time of MRI, age squared, sex, the first 10 principal components of genetic ancestry, genotyping array, and MRI center. Significant sex differences were observed, with men having a higher mean VAT of 5.0 L compared to 2.6 L in women. Lastly, we have also gathered the GWAS summary statistics on BMI and HbA1c from European populations, sourced respectively from the Genetic Investigation of ANthropometric Traits (GIANT) Consortium (https://portals.broadinstitute.org/collaboration/giant/index.php/GIANT_consortium) and the Meta-Analyses of Glucose and Insulin-related Traits Consortium (MAGIC) (https://magicinvestigators.org/) [14, 15].

### Selection criteria for instrumental variables

The genetic variants were extracted using association thresholds of *P* < 1e-5, which are commonly used in MR analysis when there are limited SNPs available for the exposure variable. Linkage disequilibrium (LD) analysis (r^2^ < 0.1, clumping distance = 500 kb) was conducted to minimize the impact of SNP associations based on the European 1000 Genomes Project Phase 3 reference panel. We further harmonized SNPs for exposure and outcome, and palindromic effects and allelic inconsistent SNPs were removed. IVs that were strongly associated with outcomes (*P* < 5e-8) were also removed. To assess the suitability of the identified IVs for representing metabolite levels, we calculated F-statistics and excluded IVs with F-statistics < 10. The F-statistics formula employed was R^2^ (N-K-1) / [K (1-R^2^)], where R^2^ represents the explained variance of the exposure by the IVs, N represents the effective sample size, and K indicates the count of variants included in the IV model. Additionally, the PhenoScanner online platform was employed to identify and remove SNPs associated with potential confounding factors (age, BMI, height, diabetes, hypertension, and nonalcoholic fatty liver disease) (http://www.phenoscanner.medschl.cam.ac.uk) [16]. Finally, to evaluate the statistical power, we utilized the online tool available at https://shiny.cnsgenomics.com/mRnd/ [17].

### MR analysis

The MR analysis was conducted using the R software, primarily utilizing packages such as “TwoSampleMR”, “MR-PRESSO”, and “MendelianRandomization”. To determine the causal effects of blood metabolites and VAT, five commonly used MR methods were employed: inverse variance weighted (IVW), weighted median, simple mode, weighted mode, and MR-Egger regression analysis. The fixed-effects IVW method, which combines Wald ratios for each SNP to calculate a pooled estimate, was used as the primary method. The other methods were used as additional measures to support the findings. False Discovery Rate (FDR) correction was employed to control for false positives in multiple testing. A statistically significant correlation was defined as having an estimated causal effect with FDR < 5%. Blood metabolites with an ***P*** value < 0.05, but not reaching the FDR threshold, were deemed to potentially have a causal effect. Additionally, the causal relationship between identified metabolites with sex-stratified VAT was also explored. Heterogeneity between IVs was quantified using Cochrane’s Q test. If ***P*** <​ 0.05, indicating the presence of heterogeneity, the random-effects IVW was used instead of the fixed-effects IVW [18]. The intercept of MR Egger regression was examined to assess the presence of underlying pleiotropy, with a *P* < 0.05 suggesting the directional pleiotropy. The MR-PRESSO test was employed to identify and quantify potential pleiotropic effects, detect outliers that could impact the study outcomes, and assess improvements after their removal. Leave-one-out analysis was also conducted to evaluate the influence of potentially significant IVs. Lastly, to unveil the potential vertical pleiotropic pathways of the identified blood metabolites, we conducted multivariable MR (MVMR) analyses, including MVMR-IVW, MVMR-Egger, and MVMR-Median, to estimate the direct causal effects of these blood metabolites on VAT after adjusting for BMI and HbA1c. The parameter settings were consistent with those of univariable MR analysis.

### Metabolic pathway enrichment analysis

Based on the identified blood metabolites with ***P*** value < 0.05, MetaboAnalyst 5.0 (https://www.metaboanalyst.ca/), an intuitive online tool specifically designed for streamlined metabolomics data analysis, was used to conduct metabolic pathway analysis [19, 20].

### Evaluation of genetic correlation and directionality

Genetic correlation between the exposure and outcome in MR analysis may lead to a violation of cause-effects [21, 22]. Despite excluding SNPs linked to VAT when choosing IVs, irrelevant SNPs might still influence VAT presence. LDSC is a statistical method used to analyze genetic correlation. By leveraging linkage disequilibrium information in the genome, it can assess the genetic correlation between two traits, such as disease and gene expression [21, 23]. To confirm that the causal effects are not muddled by shared genetics, LDSC was used to assess the genetic correlation between metabolites and VAT [24]. Furthermore, the MR Steiger test was conducted to address the potential bias arising from reverse causality [25].

### Reverse MR analysis

Based on the same criteria, we conducted a reverse MR analysis using VAT-associated SNPs as IVs to investigate if VAT accumulation causally affected the blood metabolites identified above.

## Results

### Selection of instrumental variables

After conducting a rigorous quality control process, we have identified 10,635 SNPs linked to 486 blood metabolites as IVs, ranging from 3 to 505, to explore the causal relationship between metabolites and VAT. F-statistics for each SNP were all over 10, suggesting no weak IVs were employed. Comprehensive information for each SNP was presented in Supplementary 1.

### Results of MR analysis between blood metabolites and VAT

**Fig. 1:**
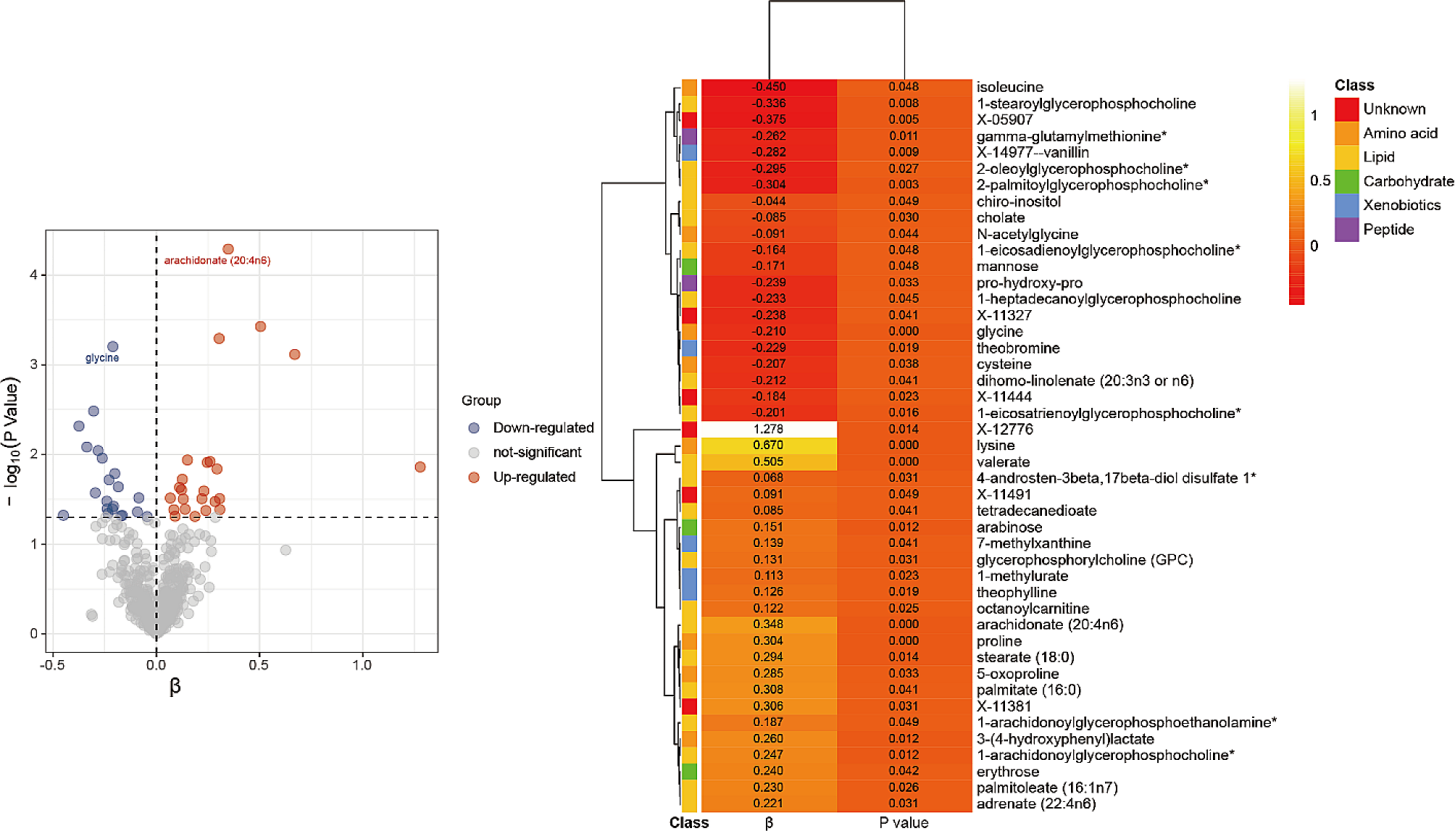
Causal relationships between forty-five metabolites and VAT

**Fig. 2:**
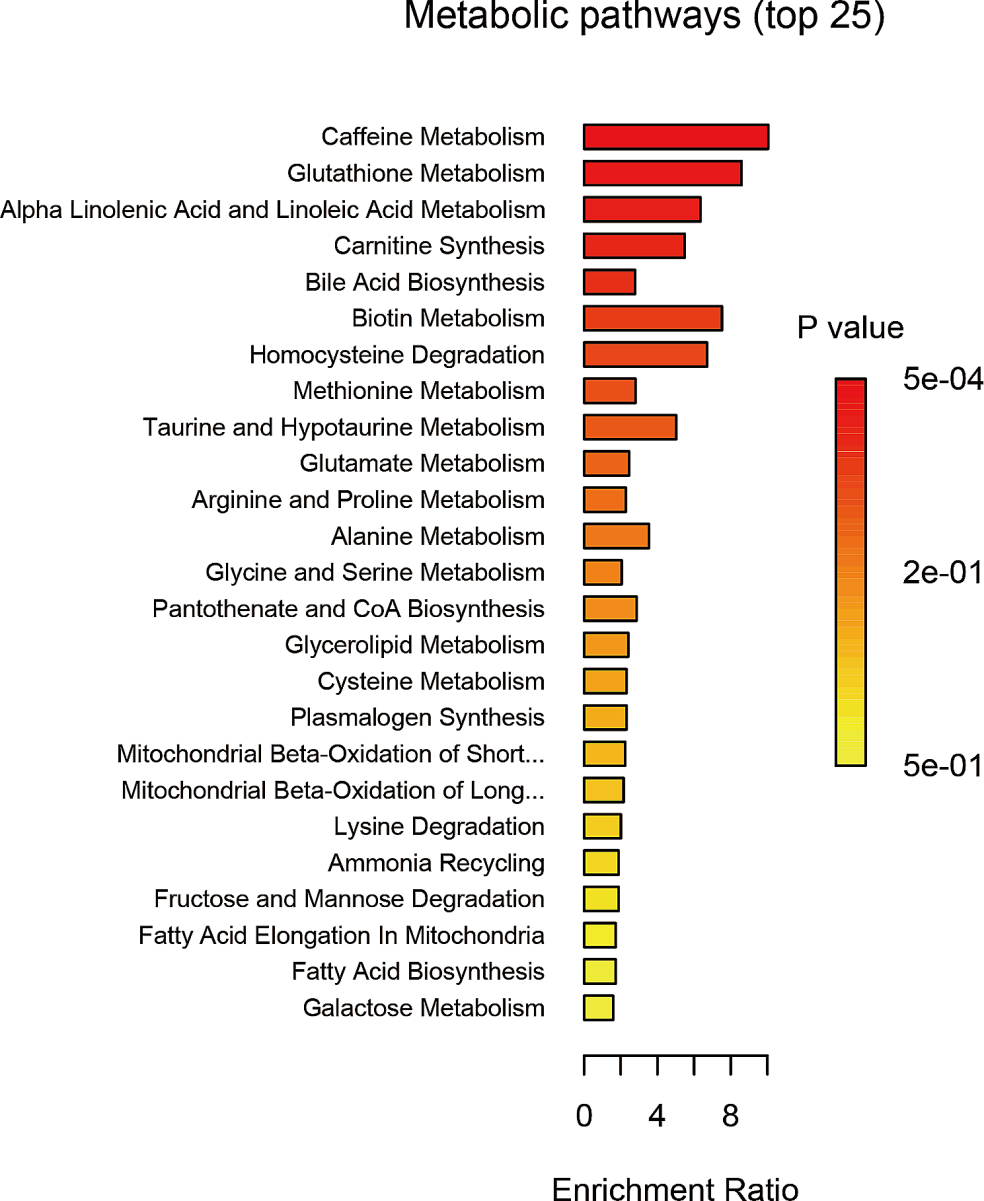
Results of pathway enrichment analysis of forty-five metabolites

The comprehensive analysis results of 486 blood metabolites and VAT was described in Supplementary 2. The IVW analysis identified a total of forty-five metabolites associated with VAT accumulation (*P* < 0.05). Among them, six metabolites remained chemically unknown. The remaining metabolites were assigned to eight amino acids, three carbohydrates, twenty-one lipids, two peptides, and five xenobiotics (Fig. 1). Based on the pathway analysis conducted using MetaboAnalyst for the identified blood metabolites, the results indicated that the top twenty-five ranked metabolic pathways are caffeine metabolism, glutathione metabolism, alpha linolenic acid and linoleic acid metabolism, and so on (Fig. 2).

**Fig. 3:**
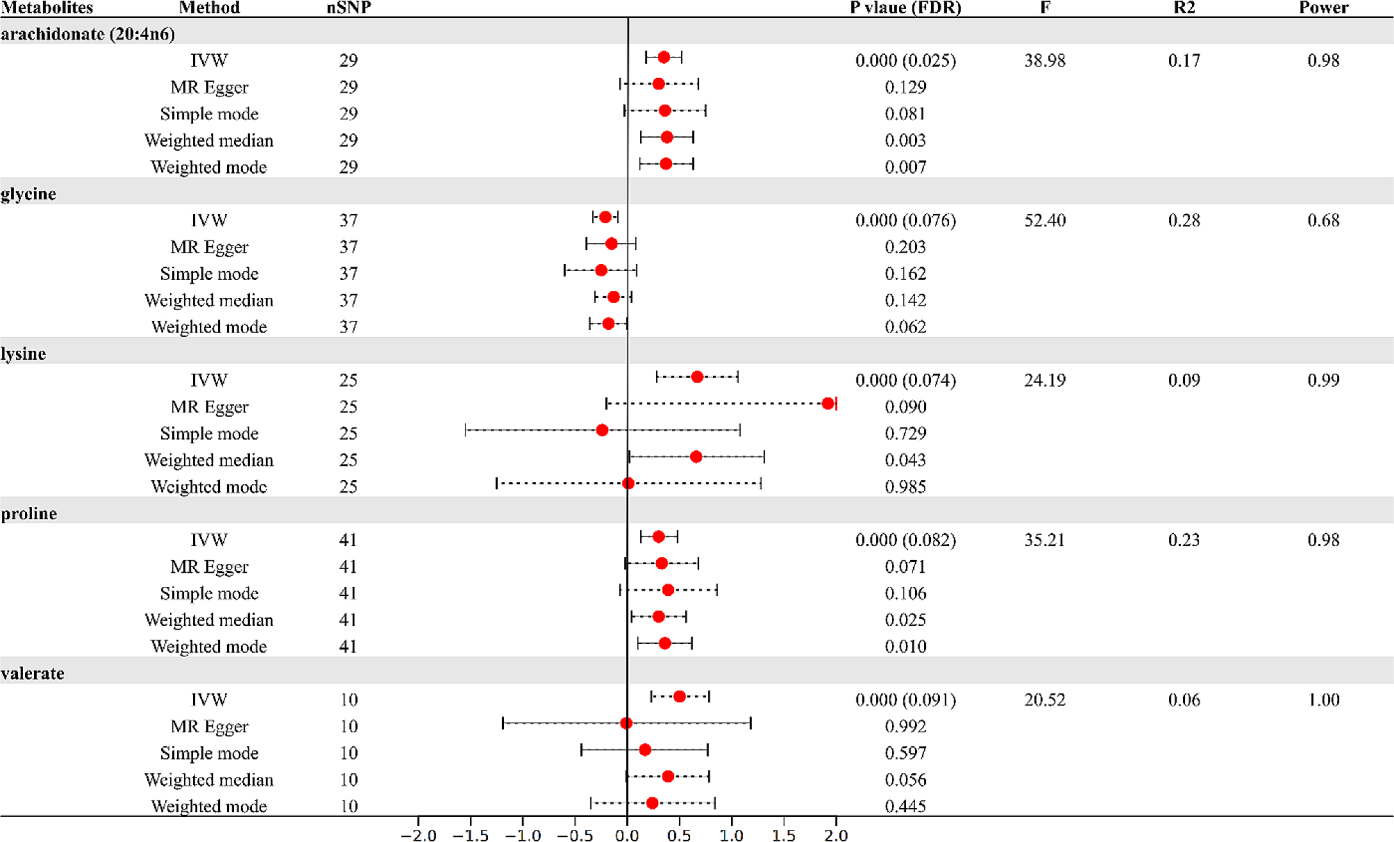
Causal relationships between five metabolites and VAT

Further after FDR correction, the IVW MR estimation identified a significance positive correlation between arachidonate (20:4n6) and the accumulation of VAT (β = 0.35, 95%CI:0.18–0.52, *P* < 0.001; FDR = 0.025). We also identified four metabolites with obvious potential causal relationship (FDR < 0.10). Higher levels of lysine (β = 0.67, 95%CI: 0.28–1.06, *P* < 0.001; FDR = 0.074), proline (β = 0.30, 95%CI:0.13–0.48, *P* < 0.001; FDR = 0.082), and valerate (β = 0.50, 95%CI:0.23–0.78, *P* < 0.001, FDR = 0.091) might also result in the accumulation of VAT. On the other hand, the presence of glycine (β=-0.21, 95%CI: -0.33–0.09), *P* < 0.001, FDR = 0.076) suggested a protective effect against the accumulation of VAT (Fig. 3). MR Egger, weighted median, simple mode, and weighted mode also demonstrated a trend of causal relationships. Supplementary 3 displayed scatterplots demonstrating the causal relationship between metabolites and VAT accumulation. Cochrane’s Q test suggested there is no indication of heterogeneity among SNPs associated with blood metabolites in predicting VAT accumulation (*P* > 0.05) (Table 1). MR-Egger regression intercept also showed no risk discrepancy due to unbalanced pleiotropy related to VAT accumulation (*P* > 0.05) (Table 1). The MR-PRESSO test further confirmed the reliability of our results (*P* > 0.05) (Table 1). Lastly, leave-one-out analysis, as shown in Supplementary 3, did not find any influential SNPs affecting the overall effect estimate.

**Table 1:** The results of Cochrane’s Q, MR-Egger intercept, and MR-PRESSO

Metabolites	Group	Q_IVW_	*P*	Egger intercept	*P*	MR-PRESSO global test
arachidonate (20:4n6)	Total	38.830	0.084	0.001	0.768	0.134
	Female	43.163	0.034	-0.004	0.470	0.051
	Male	32.222	0.266	0.006	0.242	0.324
glycine	Total	40.928	0.263	-0.002	0.571	0.290
	Female	29.018	0.789	0.001	0.804	0.829
	Male	44.190	0.164	-0.003	0.421	0.166
lysine	Total	29.104	0.216	-0.012	0.251	0.217
	Female	27.727	0.272	-0.011	0.453	0.266
	Male	21.492	0.610	-0.013	0.354	0.612
proline	Total	51.642	0.103	-0.001	0.847	0.136
	Female	57.891	0.033	0.000	0.901	0.055
	Male	36.315	0.637	-0.001	0.666	0.677
valerate	Total	15.489	0.078	0.011	0.399	0.114
	Female	15.366	0.081	0.013	0.504	0.108
	Male	7.494	0.586	0.007	0.617	0.647

### Results of MR analysis between blood metabolites and sex-stratified VAT

**Fig. 4:**
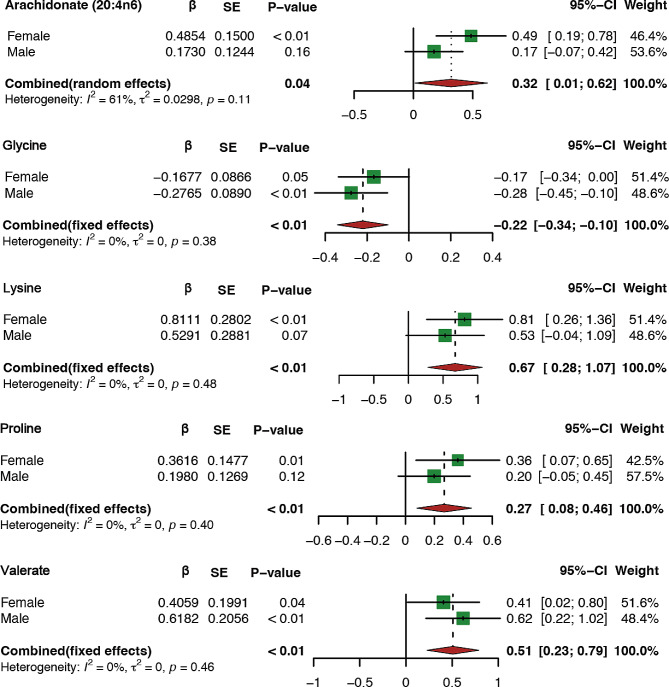
Causal relationships between five metabolites and sex-stratified VAT

We further investigated the relationship between above five blood metabolites and VAT stratified by sex (Fig. 4). Our analysis showed consistent trends between different sexes for most blood metabolites. However, the association between arachidonate (20:4n6), lysine, proline, valerate and VAT accumulation was statistically different in females (*P* < 0.05), while the associations between valerate, glycine and VAT accumulation were statistically different in males (*P* < 0.05).

### MVMR analysis

**Fig. 5:**
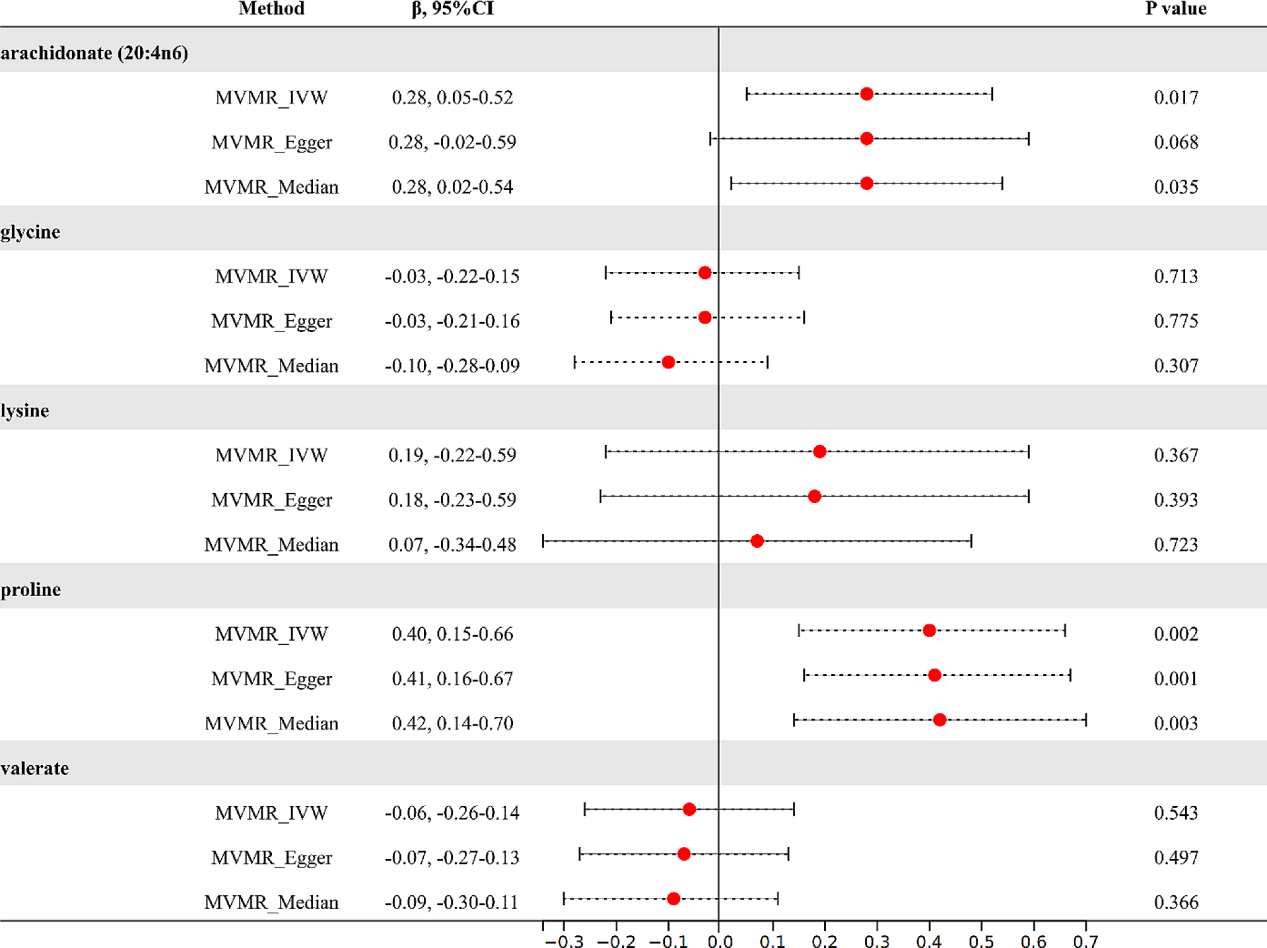
Results of MVMR analysis

We further employed a multivariable MR analysis to estimate the direct effects of these five metabolites on the accumulation of VAT (Fig. 5). Based on the MVMR_IVW results, the effect of genetically predicted arachidonate (20:4n6) (β = 0.28, 95%CI: 0.05–0.52, *P* = 0.017) and proline (β = 0.40, 95%CI: 0.15–0.66, *P* = 0.002) on VAT remained after accounting for BMI and HblA1c. The causal inference was further supported by consistent direction and magnitude from the results of MVMR_Egger and MVMR_Median model.

### Results of MR analysis at genome-wide significance threshold (5e-8)

**Fig. 6:**
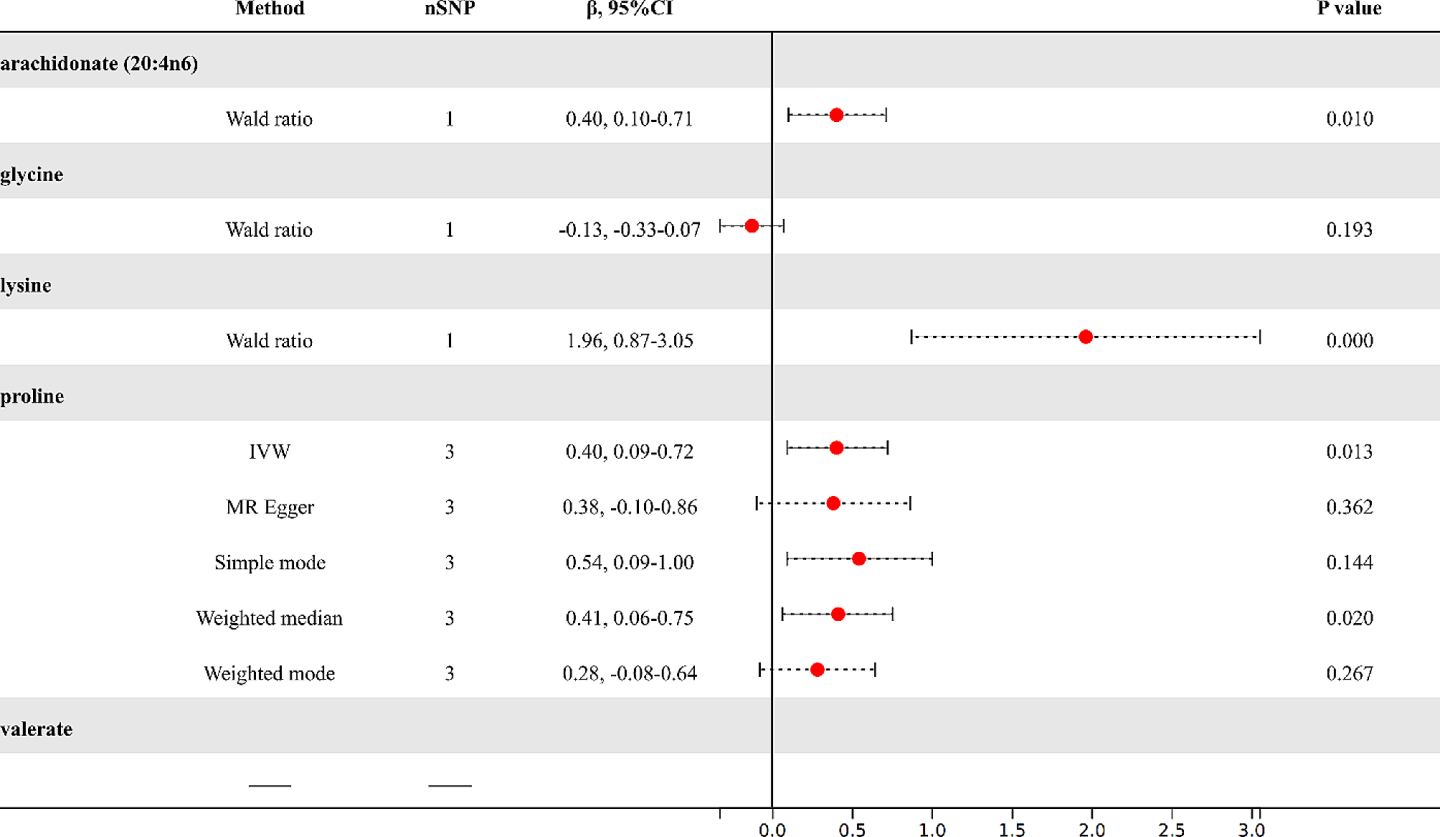
Results of MR analysis at genome-wide significance threshold (5e-8)

Upon further refinement based on genome-wide significance thresholds (5e-8) with LD analysis (r^2^ < 0.001, clumping distance = 10,000 kb), we selected IVs for five blood metabolites (Fig. 6). arachidonate (20:4n6), glycine, and lysine each had one SNPs as IVs; proline had three SNPs as potential IVs. However, for valerate, no suitable IVs were found. Further IVW analysis indicated that the increased levels of arachidonate (20:4n6) (β = 0.40, 95%CI: 0.10–0.71, *P* = 0.010), lysine (β = 1.96, 95%CI: 0.87–3.05, *P* < 0.001), and proline (β = 0.40, 95%CI: 0.09–0.72, *P* = 0.013) remain as the risk factors for the augmented accumulation of VAT.

### Evaluation of genetic correlation and directionality

To determine if the association between metabolites and VAT is due to shared genetic structure, we conducted LDSC analysis (Supplementary 3). Based on the total VAT, the results indicate no significant genetic correlation between arachidonate (20:4n6), glycine, lysine, valerate, and VAT accumulation. However, there may be a genetic correlation between VAT and proline (Rg = 0.190 *P* = 0.011). It is important to acknowledge that the limited sample size of the metabolites may have compromised the statistical power of our analysis. Steiger test indicated no reverse causation bias in the identified causal relationships (*P* < 0.05) (Supplementary 3). Finally, reverse MR analysis did not find evidence of the causal relationship between VAT accumulation and the five blood metabolites mentioned above (Supplementary 2). For other potential causal metabolites, reverse MR analyses results indicated that suggest that there is a bidirectional causal relationship between the accumulation of VAT and arabinose, N-acetylglycine, 1-stearoylglycerophosphocholine, 2-palmitoylglycerophosphocholine*, chiro-inositol.

### Discussion

To our knowledge, this is the first MR study to assess the causal role of human blood metabolites in visceral obesity.

Obesity is a multifactorial disease with significant heterogeneity. It manifests in various phenotypes, which can be either metabolically unhealthy or healthy [26]. Metabolomics, a high-throughput, and unbiased profiling technique, enables the simultaneous quantification of a wide range of small-molecule metabolites within biological systems [27]. By analyzing the metabolome, which represents the end-products of cellular processes, metabolomics offers a unique opportunity to uncover the intricate metabolic alterations associated with obesity [28]. We have identified that the metabolic pathways primarily enriched in these metabolites include caffeine metabolism. Current research consistently suggests a strong correlation between caffeine metabolism and obesity, including anti-inflammatory and antioxidant effects that promote fat oxidation, increase energy metabolism, and suppress appetite [29–31]. Previous non-targeted metabolomics study focusing on the obese people and animals also revealed that caffeine and caffeine-related metabolism pathways were the most prominent metabolic pathways [32, 33]. Additionally, previous studies have suggested that some of these blood metabolites, such as arachidonate (20:4n6) [34], glycine [35], palmitoleate [36], theophylline [37], and so on, might be involved in the pathogenesis of obesity and serve as biomarkers for obesity. However, most of these studies have focused on general obesity measures such as BMI, while the distribution of body adipose tissue plays a crucial role in obesity and its associated health risks. By leveraging previous GWAS studies, we can more accurately identify the harmful obesity phenotype, specifically visceral obesity. Furthermore, based on the advantages MR studies, we have further elucidated the pathogenic and protective effects of these blood metabolites on the accumulation of VAT. This additional information provides a more comprehensive understanding of the role of these blood metabolites in visceral obesity and related health outcomes.

After multiple testing correction, arachidonate (20:4n6) has been implicated in VAT accumulation. Arachidonate is a crucial essential fatty acid in the human body, and it is the most abundant and widely distributed polyunsaturated fatty acid. The metabolic products of arachidonate include a series of prostaglandins and leukotrienes, which are highly active inflammatory mediators. Studies have shown that it has a pro-inflammatory effect in the inflammatory microenvironment of 3T3-L1 adipocytes induced by lipopolysaccharides [38]. Additionally, four other blood metabolites (glycine, lysine, proline, and valerate) were identified as having obvious potential causal relationship (FDR < 0.10). Glycine, the amino acid with the lowest molecular weight, shows lower circulating levels in metabolic disorders related to obesity, type 2 diabetes (T2DM), and non-alcoholic fatty liver disease (NAFLD), and increasing glycine levels can inhibit these disorders in clinical application [35]. Lysine acetylation plays a crucial role in both immune and metabolic pathways, regulating the balance of energy storage and expenditure. Current evidence suggests that lysine acetylation can modulate innate immune and metabolic pathways related to obesity and metabolic diseases [39]. Proline, playing a role in the regulation of food intake and body fat accumulation suggests its potential as a target for interventions in managing visceral obesity [40]. However, there is limited research on the relationship between valerate and VAT accumulation and obesity. Further exploration is needed in the future to better understand the potential impact of valerate on visceral obesity.

There are several limitations to this study that may impact the interpretation of the results. Firstly, the SNP used did not meet the GWAS significance threshold (5e-8). Although we relaxed the selection criteria for IVs, it was considered an acceptable threshold for blood metabolites and has been adopted in other articles [23, 41]. Secondly, most participants in our study were of European descent. While this helped to minimize population heterogeneity, it is important to validate the MR results in other populations to ensure their generalizability. Thirdly, VAT, due to its metabolic activity, could induce changes in the metabolome. We conducted a reverse MR analysis only on metabolites with potential causal relationships, and future research should delve deeper into the impact of VAT on human metabolism from this perspective. Furthermore, conducting further MR analyses, such as MR-RAPS, and reinforcing the reliability of results through replication validation based on external populations, are also necessary. Lastly, while the MR approach is excellent for causal inference, it is crucial to validate the findings from this study in well-powered randomized controlled trials.

### Electronic supplementary material

Below is the link to the electronic supplementary material.

Supplementary Material 1: The detailed characteristics of IVs

Supplementary Material 2: MR analysis results

Supplementary Material 3: The results of scatterplots, leave-one-out, LDSC, and Steiger test

## Data Availability

No datasets were generated or analysed during the current study.
