# Supplementary material for: The causal relationship between human blood metabolites and the risk of visceral obesity: a mendelian randomization analysis
Source: Lipids Health Dis. 2024 Feb 7;23:39. doi: 10.1186/s12944-024-02035-x (PMC10851536; doi:10.1186/s12944-024-02035-x)
Supplement: Supplementary file 2 — Supplementary Material 2: MR analysis results [file 12944_2024_2035_MOESM2_ESM.docx]

Scatter plots of the causal effect of five metabolites on VAT.


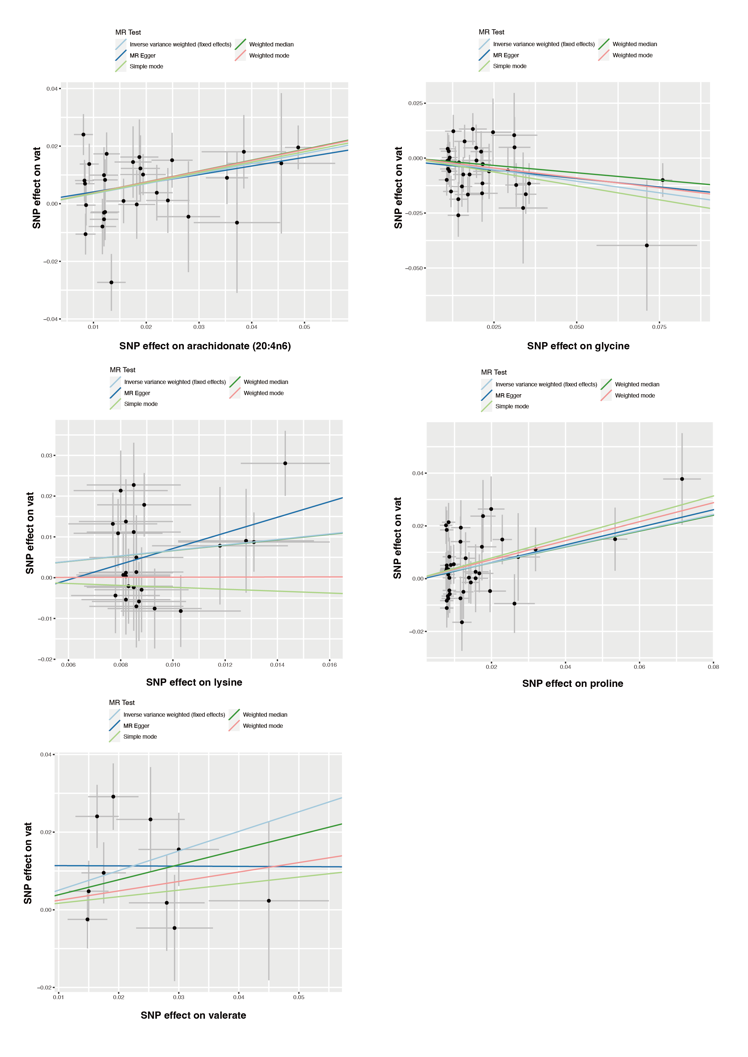


Leave-one-out method results of five metabolites on VAT.


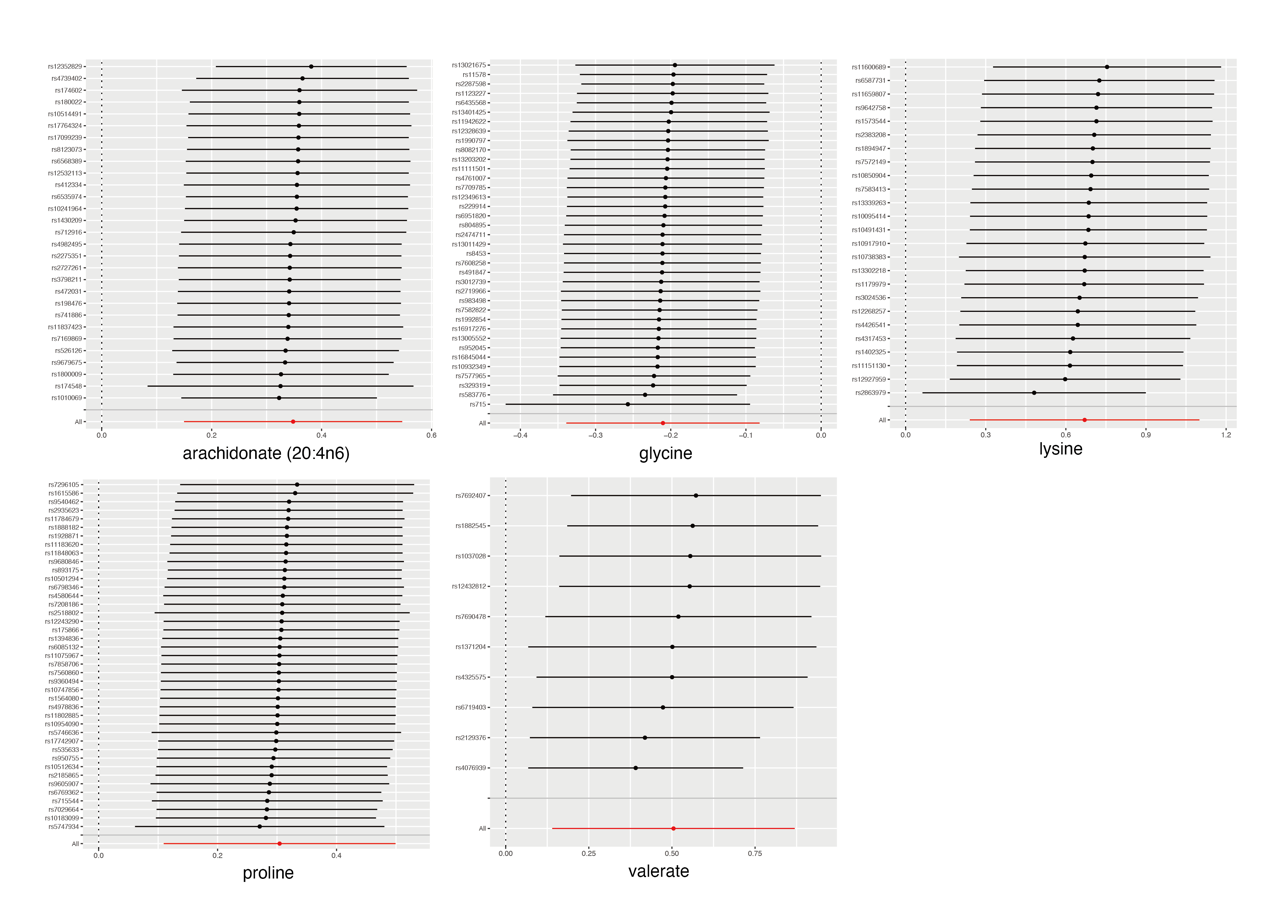


| The results of LDSC analysis and Steiger test | | | | |  |
| --- | --- | --- | --- | --- | --- |
| **Trait 1** | **Trait 2** | **Rg** | **Rg_se** | **Rg_*P*** | **Steiger*_P*** |
| arachidonate (20:4n6) | VAT (total) | 0.465 | 0.252 | 0.065 | 6.72E-209 |
|  | VAT (female) | 0.468 | 0.300 | 0.118 | 1.66E-207 |
|  | VAT (male) | 0.245 | 0.296 | 0.407 | 3.18E-217 |
| glycine | VAT (total) | -0.150 | 0.086 | 0.083 | 0 |
|  | VAT (female) | -0.248 | 0.107 | 0.020 | 0 |
|  | VAT (male) | -0.031 | 0.102 | 0.761 | 0 |
| lysine | VAT (total) | 0.517 | 0.312 | 0.098 | 1.33E-103 |
|  | VAT (female) | 0.099 | 0.356 | 0.782 | 6.69E-105 |
|  | VAT (male) | 0.550 | 0.294 | 0.061 | 9.55E-109 |
| proline | VAT (total) | 0.190 | 0.075 | 0.011 | 5.75E-293 |
|  | VAT (female) | 0.120 | 0.092 | 0.193 | 6.86E-292 |
|  | VAT (male) | 0.211 | 0.091 | 0.021 | 7.76E-304 |
| valerate | VAT (total) | 0.194 | 0.142 | 0.171 | 8.71E-39 |
|  | VAT (female) | 0.169 | 0.154 | 0.274 | 2.69E-40 |
|  | VAT (male) | 0.162 | 0.137 | 0.236 | 6.65E-41 |
